# Selective screening for inborn errors of metabolism using tandem mass spectrometry in West Kazakhstan children: study protocol

**DOI:** 10.3389/fgene.2023.1278750

**Published:** 2024-01-12

**Authors:** Gulmira Zharmakhanova, Victoria Kononets, Saule Balmagambetova, Lyazzat Syrlybayeva, Eleonora Nurbaulina, Zhanna Zhussupova, Svetlana Sakhanova, Dinmukhamed Ayaganov, Svetlana Kim, Akmaral Zhumalina

**Affiliations:** ^1^ Department of Natural Sciences, West Kazakhstan Marat Ospanov Medical University, Aktobe, Kazakhstan; ^2^ Department of Oncology, West Kazakhstan Marat Ospanov Medical University, Aktobe, Kazakhstan; ^3^ Department of General Medical Practice, West Kazakhstan Marat Ospanov Medical University, Aktobe, Kazakhstan; ^4^ Aktobe Regional Tertiary Care Center, Department of Neonatal Pathology, Aktobe, Kazakhstan; ^5^ Scientific-Practical Center, West Kazakhstan Marat Ospanov Medical University, Aktobe, Kazakhstan; ^6^ Department of Neurology, West Kazakhstan Marat Ospanov Medical University, Aktobe, Kazakhstan; ^7^ Department of Children’s Diseases No. 2, West Kazakhstan Marat Ospanov Medical University, Aktobe, Kazakhstan; ^8^ Department of Children’s Diseases No. 1 with Neonatology, West Kazakhstan Marat Ospanov Medical University, Aktobe, Kazakhstan

**Keywords:** inborn errors of metabolism, tandem mass spectrometry, selective screening, Kazakhstan, amino acids, acylcarnitines, succinylacetone

## Abstract

Data on the prevalence of most inborn errors of metabolism are still unavailable in Kazakhstan. The study aims to perform selective screening for hereditary metabolic diseases among patients aged from 1 day to 18 years in western Kazakhstan using the LC-MS/MS method, with establishing the reference values for the content of amino acids, acylcarnitines, and succinylacetone in blood samples of healthy children. Tasks: 1. To assess the burden of metabolic disorders detected by LC-MS/MS in western Kazakhstan by examination of children at clinical risk in pediatric clinics throughout the region; https://www.frontiersin.org/register?returnUrl=https://loop.frontiersin.org 2. To set the reference values of metabolites in the child population; 3. To analyze the age distribution, prevalence, and age of onset for each identified IEM, further comparing the obtained findings with those from previously published reports in other populations. Methods: To set the reference values of 51 metabolites in the child population, 750 healthy children will be included. The selective screening will be performed among 1,500 patients aged 1 day to 18 years with suspected hereditary metabolic disorders. Anticipated results: The results of selective screening will be interpreted by comparison with the reference values established. Diagnosis will be based on clinical signs, blood levels of amino acids, acylcarnitines, succinylacetone, and urine levels of organic acids and tests for gene mutations. An assessment of 37 inborn errors of metabolism frequencies in high-risk children will be performed. The research will further develop the national as selective as expanded newborn screening programs. The study was registered in clinicaltrials. gov (https://www.clinicaltrials.gov/study/NCT05910151) on 16 June 2023.

## 1 Introduction

Selective screening is an essential tool for diagnosing various types of inborn errors of metabolism (IEM). IEMs are a group of phenotypically and genotypically heterogeneous metabolic disorders caused by mutations in genes encoding enzymes of metabolic pathways or receptors. Deficiency or change in the activity of necessary enzymes or other proteins in intermediate metabolic pathways leads to the accumulation or deficiency of the corresponding metabolites in cells or body fluids, manifesting in a wide range of diseases with clinical heterogeneity, thus complicating their diagnosis (Mak et al., 2013). The classification of existing IEM is based on the biochemical properties of the substances involved (Han et al., 2015). Although these disorders are rare individually, they are numerous in the aggregate (Ozben, 2013). Many IEM do not have specific clinical signs and are difficult to diagnose using only clinical manifestations or routine laboratory tests (Champion, 2010). Most often, IEMs occur in early infancy and childhood, and the prevalence within different racial and ethnic groups is not the same. Hence, there are population differences in the incidence of IEM (Champion, 2010; Lampret et al., 2015; Shibata et al., 2018; Sarker et al., 2019). IEMs typically lead to irreversible neurological and psychological damage and/or disability or death in affected children. Early diagnosis of IEM can significantly reduce the risk of death and may prevent long-term neurological complications (Lampret et al., 2015; Dietzen et al., 2016). The purpose of NBS (newborn screening) is to presymptomatically identify infants with congenital conditions so that treatment can be started as early as possible and prevent or mitigate the long-term consequences of the condition. The expanded newborn screening program increased the screening panel of disorders from six (6) to more than twenty-eight (Pitt, 2010). IEMs represent the largest category of hereditary diseases amenable to causal therapy (van Karnebeek and Stockler-Ipsiroglu, 2014).

Selective IEM screening is usually performed for patients with clinical symptoms, positive results of routine laboratory tests, or having a family history suggesting a metabolic disorder. The methods most commonly used for metabolite detection have included gas chromatography-mass spectrometry (GC-MS) for organic acids, ion exchange chromatography-post column derivatization for amino acids (AAs), and, lately, liquid chromatography-tandem mass spectrometry (LC-MS/MS) for the analysis of AAs, acylcarnitines (ACs), and succinylacetone (SuAc) (Chace, 2009; Lampret et al., 2015; Gelb et al., 2022; Kaysheva et al., 2023). Currently, widely used technologies based on LC-MS/MS allow for simultaneously determining several metabolites, such as AAs, ACs, and SuAc, from a small amount of a biological sample (Gaugler et al., 2017). However, involved laboratories first should establish age reference cut-off ranges for each analyte for each population before patient screening/diagnosis, as cut-offs depend on various factors such as genetic background, geographic location of the population, diet, gender, age, etc. (Dietzen et al., 2016; Dogan et al., 2017; Yang et al., 2018; Sarker et al., 2019). The expansion of newborn screening beyond phenylketonuria (PKU) has occurred mainly due to the introduction of tandem mass spectrometry, which allows testing for multiple metabolic conditions from a single drop of blood (Kruszka and Regier, 2019).

At present, newborns are screened using LC-MS/MS as part of expanded newborn screening (ENBS) programs in most high-income and some low/middle-income countries (LMICs) (Sarker et al., 2019). Utilizing relatively simple tests, including detecting AAs, SuAc, and ACs in dry blood spots on filter paper, tandem mass spectrometry allows rapid screening and diagnosis of IEM (Han et al., 2015). The prognosis of patients with diseases targeted by ENBS has improved markedly in countries implementing the screening system (Therrell et al., 2014; Landau et al., 2017; Shibata et al., 2018). The NBS panels for each country, and sometimes for different regions within the same country, vary depending on the prevalence of each disorder in the population and the health system financing policy (Han et al., 2015; Hassan et al., 2016). Many LMICs do not yet have their national NBS programs. Meanwhile, local data on IEM incidence and outcomes can be used to persuade health officials to prioritize screening in healthcare expenses (Therrell and Padilla, 2018). Data on the prevalence of each IEM can serve as an essential tool to evaluate the cost-effectiveness of screening. Therefore, selective screening is the first step in projects to study the prevalence of IEM in developing countries experiencing economic difficulties. Developing countries face challenges in implementing and expanding their IEM screening programs, including financial issues, medical and logistical support, public education, policy development, program evaluation, and sustainability providing (Padilla et al., 2010; Hassan et al., 2016). LC-MS/MS can detect and quantify many metabolites in a single blood spot to diagnose amino acid disorders, organic acidemias, fatty acid oxidation disorders, and urea cycle disorders. MS/MS is expanding to implementing NBS programs for IEM and selective screening of children of different ages. The results from these expanded NBS programs provided information on the prevalence of these diseases in the United States (Therrell et al., 2014; Landau et al., 2017), some countries in Europe (Scolamiero et al., 2015; Messina et al., 2018; Lampret et al., 2020; Loeber et al., 2021), and Asia (Yunus et al., 2016; Dogan et al., 2017; Yang et al., 2020; Deng et al., 2021).

In Kazakhstan, MS/MS in metabolic screening programs is not yet developed due to the high cost of equipment and consumables and the lack of special screening centers and specialists. Data on the prevalence of most IEM, except phenylketonuria (Loeber et al., 2021), are still unavailable in Kazakhstan. The data obtained in this study can provide a comparative analysis of the prevalence of IEM in Asia and establish reference values for AAs, ACs, and SuAc concentrations in the blood of newborns in Kazakhstan. Reference interval data for acylcarnitines, succinylacetone, and amino acids obtained from the population of newborns in western Kazakhstan will be the first step in developing a national IEM diagnostic program.

The efficacy and risks of expanded newborn screening for IEM require careful evaluation before it can be accepted as a mandatory national program. The lack of epidemiological data on the incidence of IEM is one of the reasons for the delay in the development of proper population-based newborn screening for IEM using the LC-MS/MS method in Kazakhstan. Currently, NBS is being conducted in Kazakhstan for two hereditary diseases - phenylketonuria (PKU) and congenital hypothyroidism, the most represented screening diseases in most countries (Therrell and Padilla, 2018). This nationwide NBS program based on biochemical tests started in 2007 (Loeber et al., 2021), long after NBS was introduced in the United States in 1963 (Guthrie and Susi, 1963). Besides, in Kazakhstan, before the present study, state-funded MS/MS selective screening programs were implemented as sporadic pilot projects in some regions of the country without completion. Reports on these projects have not been published in peer-reviewed journals. As MS/MS neonatal screening is not mandatory in Kazakhstan, there are no uniform technical specifications for IEM screened by MS/MS in pilot studies. Hence, data on the frequency of IEM at the national level are not presented. For a country with no mandatory ENBS MS/MS program, selective screening can become essential for diagnosing IEM. The present research aims to develop and validate the LC-MS/MS method for simultaneously determining 51 metabolites in dry blood spots for IEM screening, as well as clarifying age-related ranges for AAs, ACs, and SuAc in Kazakhstan children. It is commonly accepted that children need to use reference populations that reflect changes associated with growth and development (Dogan et al., 2017). Therefore, one of the objectives of this study is to set reference intervals for the concentration of amino acids, succinylacetone, and acylcarnitines in dry blood spots for different age groups in the range from 1 day to 18 years. Owing to historically established social-cultural traditions supported by the legislation, consanguineous marriages are practically absent in Kazakhstan. This distinguishes Kazakhstan from many Asian and European countries, where consanguineous marriages are a real problem and, according to researchers, they are one of the reasons for the relatively high incidence of certain groups of IEM identified during selective newborn screening (Nagaraja et al., 2010; Al Riyami et al., 2012; Golbahar et al., 2013; Selim et al., 2014; Hassan et al., 2016; Demirelce et al., 2020; Gayduk et al., 2022; Magdy et al., 2022).

Accordingly, *the research hypothesis* is the following. Given the mentioned above social-cultural features of the Kazakh’s way of life, the obtained frequencies of different IEM are expected to be lower than in other countries practicing historically approved consanguineous marriages. Thus, *the study goal* is to perform selective screening for hereditary metabolic diseases among patients aged from 1 day to 18 years in western Kazakhstan using the LC-MS/MS method, with establishing the reference values for the content of amino acids, acylcarnitines, and succinylacetone in blood samples of healthy children.

Tasks:

1. To assess the burden of metabolic disorders detected by LC-MS/MS in western Kazakhstan by examination of children at clinical risk in pediatric clinics throughout the region;
2. To set the reference values of metabolites in the child population;
3. To analyze the age distribution, prevalence, and age of onset for each identified IEM, further comparing the obtained findings with those from previously published reports in other populations.

## 2 Methods and analysis

### 2.1 Study design and timing

**FIGURE 1:**
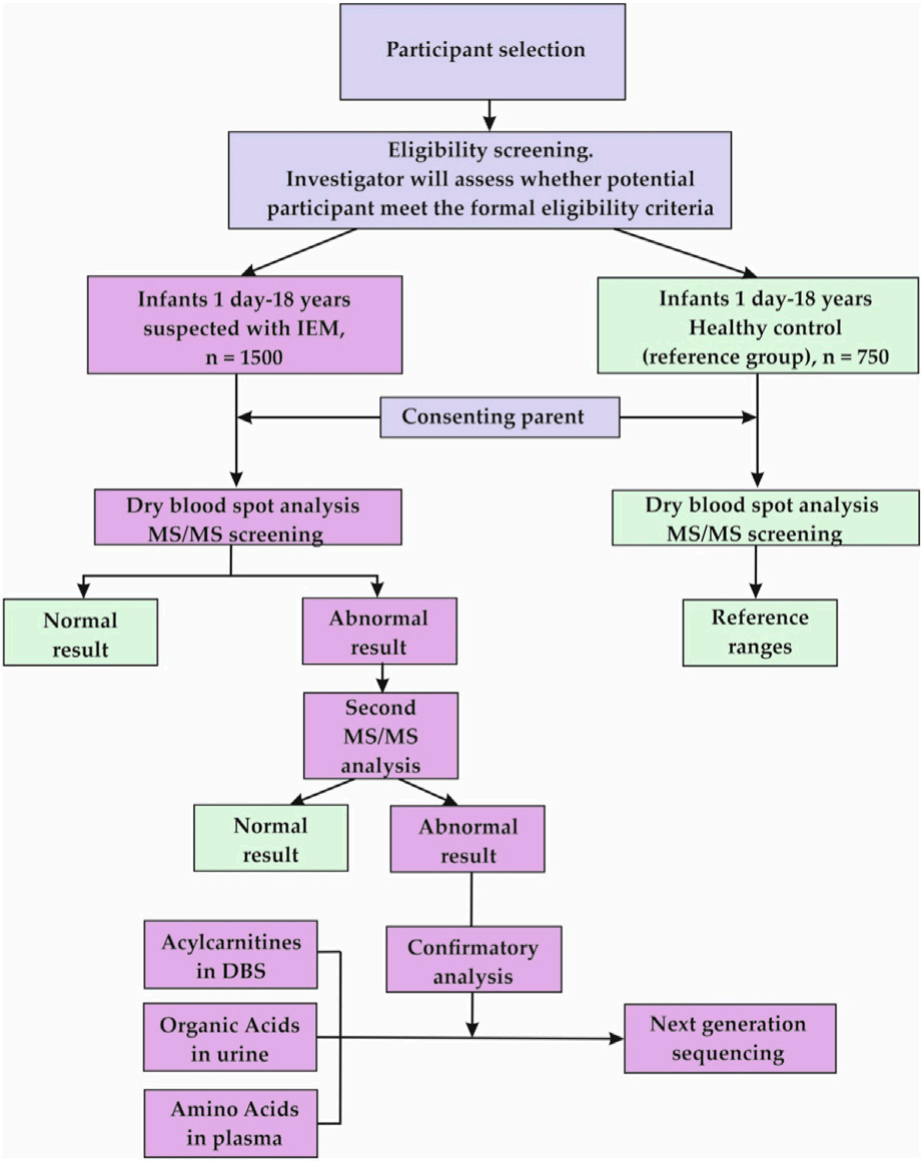
Flowchart of the study.

This observational study, uses a cross-sectional design due to its screening nature. The data for the present research will be derived from a selective LC-MS/MS IEM screening of 1,500 clinical-risk children aged 1 day to 18 years and 750 healthy controls of the same age to set reference values for 15 AAs, 35 ACs, and SuAc. The main points of the research are presented in Figure 1 (Flowchart of the study.)

The project timing is calculated for 3 years, 2022–2024. The recruitment of healthy children for establishing reference values started on October 2022, and the overall data collection will last until August 2024 (recruitment of high-risk children began in 2023).

### 2.2 Selection of subjects

#### 2.2.1 Sample size estimation

When calculating the sample size for the upcoming project on selective screening, we relied on published similar studies. Researchers pointed out the following essential factors contributing to the sample size calculation: Daniel’s formula for prevalence studies in case of expanded screenings, the reported frequencies of different IEM within the studied populations, and the number of infants born in the country during the period of interest (Yoon et al., 2005; Golbahar et al., 2013; Hassan et al., 2016; Shibata et al., 2018; Sarker et al., 2019; Manta-Vogli et al., 2020). Notably, many researchers corrected their calculations, considering the situation with consanguineous marriages in their country.

According to the review by Shibata N et al., the IEM overall detection frequency was 3.0% (1.2% in Japan and 9.2% in other Asian countries) (Shibata et al., 2018). Yoon et al. reported 5.4% IEM among South Korean annual births (Yoon et al., 2005). Of these data on IEM frequency throughout Asian countries, Mongolian values (1.6%) have attracted our attention due to the similar nomadic history and way of life where consanguineous marriages were strictly forbidden (Shibata et al., 2018). Along with that, the Mongolian index does not include all types of IEM except the most distributed.

Based on mentioned factors, we requested data on the number of childbirths across the four provinces (oblasts) of western Kazakhstan within 3 years before the project started. The average rate of infants born alive annually ranged between 69–73,000 (https://stat.gov.kz/en/industries/social-statistics/demography/publications/). Thus, we have decided to build on the IEM average frequency of 2%–2.5% and determined the expected sample size of 1,500 children for selective screening with 750 healthy controls.

#### 2.2.2 Study population. inclusion and exclusion criteria

**FIGURE 2:**
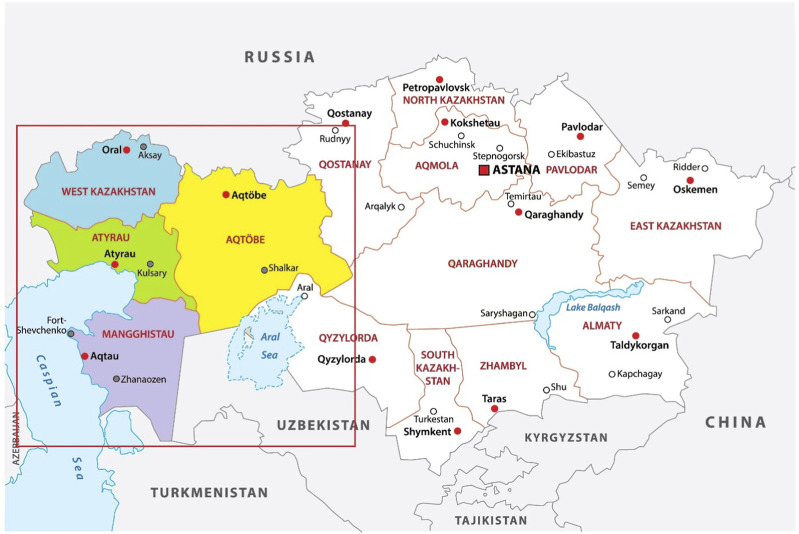
Sites for data collection throughout western Kazakhstan.

In this research, we are consecutively recruiting a sample of eligible 2,250 children across the region (1,500 children aged 1 day to 18 years suspected of IEM and 750 healthy children to set the reference values). Western Kazakhstan is divided into four regions (provinces, oblasts) according to the administrative division: Aktobe, West Kazakhstan, Atyrau, and Mangystau (Figure 2).

Children from the western Kazakhstan population with suspected metabolic disorders are examined for congenital metabolic disorders and referred by primary care neonatologists and pediatric consultants between October 2022 and August 2024 based on their clinical symptoms associated with metabolic disorders. The study includes newborns and children being treated in maternity wards and pediatric clinics of 7 pediatric hospitals in the region. Patients with suspected IEM from all sites are referred to the Regional Tertiary Care Center or the Mother and Infant Health Center in Aktobe, or their samples are sent from other involved hospitals. Patient information is obtained from the Individual Registration Cards (IRCs) completed by study participants. The completed questionnaires are checked by the researchers. In case of improper collection, data must be removed from the study if it is impossible to check adequately. The IRC sample and other supplementary materials are placed in the *clinical. trials.gov* Register (https://www.clinicaltrials.gov/study/NCT05910151) and a publicly available repository (https://osf.io/cmrh7/).

To set reference values of 15 AAs, 35 ACs, and SuAc, healthy participants without any disease are included in the study. A total of 750 healthy male and female children aged 1 day to 18 years are recruited based on random selection. Participants will be balanced by gender and place of residence. Considering the distribution of urban and rural populations in western Kazakhstan, approximately half of them will be selected in urban areas, the other half in rural areas. A summary of the age, gender, and geographic distribution of study participants will be presented in Table 1.

**TABLE 1:** Demographic data to be collected on study participants.

Variables	Selective screening (n = 1,500)	Healthy children (n = 750)
Age in days, years (median, range)		
Weight in grams, kilograms (median, range)		
Gender		
Male, n		
Female, n		
Geographic distribution		
Aktobe province		
Atyrau province		
West Kazakhstan province		
Mangystau province		
Urban population		
Rural population		

Screened children with positive first-level LC-MS/MS test results will be referred for second-level screening.

#### 2.2.3 Criteria for inclusion of healthy children in the study (to set reference values)

Pediatricians examine all children in this study to ensure they do not suffer from any disorder or chronic disease. Healthy male and female newborns born after an uncomplicated pregnancy and vaginal delivery should have a body weight of 2,500–4,000 g, gestational age of 37–42 weeks, and an APGAR score greater than 7 in 10 min after birth. None should be diagnosed with birth asphyxia, defined as an Apgar score ≤6 at 5 min. All newborns must be breastfed, and their mothers must be healthy persons aged between 24 and 36. They must not have any food restrictions (vegetarian, vegan, etc.). Echograms of the placenta and fetus, as well as laboratory tests, should be normal throughout pregnancy.

#### 2.2.4 Criteria for inclusion in the selective screening for IEM

Children from 1 day to 18 years of age will be enrolled for selective screening for IEM if one of the main criteria or two or more additional criteria (symptoms) are identified. The inclusion criteria for IEM selective screening are presented in Table 2.

**TABLE 2:** Criteria for inclusion in the selective screening for IEM.

Main criteria (symptoms)	Sudden deterioration in the clinical condition of the child after a period of normal development (days, weeks, months): Acute metabolic encephalopathy lethargy (coma) seizures resistant to antiepileptic therapy
	Hepatomegaly (hepatosplenomegaly)
	Metabolic acidosis with increased anion gap
	Multiple fractures
	Child mortality in the family from diseases with similar symptoms
Additional criteria (symptoms)	Treatment-resistant seizures
	Abnormal muscle tone: dystonia, hyperkinesis, hypotension
	Speech retardation
	Mental retardation of unknown cause
	Cardiomyopathy
	Tachypnea
	Frequent spitting up (vomiting)
	Osteoarticular anomalies (joint stiffness, chest deformity, rickets-like changes)
	Hernias (umbilical, inguinal-scrotal)
	Persistent or recurrent hypoglycemia
	Metabolic alkalosis
	An increase in ketone bodies in the blood and (or) urine
	Hyperammonemia
	An increase in the level of liver enzymes (ALAT, AST) more than 1.5 times
	Increase in the level of creatine phosphokinase (CPK) by more than 2 times
	Decrease in the level of alkaline phosphatase (AP) below the age norm
	Imaging or electrophysiological examinations indicating a metabolic disorder
	Leukopenia
	Thrombocytopenia
	Abnormal odor of urine, body, earwax, any unusual odor
	Hair growth disorders, alopecia
	Ophthalmic anomalies
	Unusual appearance, dysmorphic features
	History of death of a previous sibling of unknown cause
	Parents’ consanguinity
	Positive family history with metabolic disorders

Exclusion criiteria:

Patients having the following conditions will be excluded:

1. Perinatal brain injury,
2. Brain injury,
3. Infections of the central nervous system,
4. Toxicological diseases,
5. Tumors,
6. Chromosomal abnormalities,
7. Symptoms specified in the Inclusion criteria, but with a confirmed diagnosis of any disease other than Amino acid disorders (AAD), Fatty acid oxydation disorders (FAOD), or Organic acidemias (OA).

### 2.3 Methods and procedures

#### 2.3.1 Mass spectrometry analysis. specimen collection and storage

Neonatal whole blood samples are taken from high-risk and healthy infants no earlier than 3 h after feeding by heel prick with a heel stick. Five drops of whole blood (∼75 µL each) are put onto Guthrie cards, Ahlstrom 226 filter paper, PerkinElmer 226 Five-Spot Card (PerkinElmer Health Sciences, Greenville, United States) to form dry blood spots (DBSs) for LC-MS/MS analysis. The sample must be taken before transfusion therapy or extracorporeal membrane oxygenation. If the sample was not taken before the administration of transfusion agents, blood should be taken no earlier than 48–72 h after the transfusion.

Whole blood samples from older children at high-risk and healthy children are collected after 4-hour fasting using a standard venipuncture method. The DBS card is prepared by applying five drops of whole blood (each ∼75 μL) to the Guthrie cards, Ahlstrom 226 filter paper, PerkinElmer 226 Five-Spot Card (PerkinElmer Health Sciences, Greenville, United States).

Samples must be dried for 4 h at room temperature and then stored at 4°C in individually labeled zippered plastic bags with desiccants or other sealed containers until analyzed by LC-MS/MS by standards of the Institute of Clinical and Laboratory Standards (CLSI, 2013). Samples must be sent to the laboratory within 5 days. In case of long-term storage of samples, it will be carried out at a temperature of −20°C. Cut-off values will be set according to the manual for the Neobase2 TM Non-derivatized MSMS kit (PerkinElmer, Wallac Oy, Turku, Finland). They will be adjusted over time as the number of samples increases.

If metabolite concentrations in dry blood spot samples exceed the cut-off values, the LC-MS/MS test will be repeated using the same dried blood spot sample the next day. If the test results show persistent abnormal results, the child will be further examined. Blood and urine are used to confirm the diagnosis. Diagnosis is confirmed by urine organic acid analysis, plasma amino acid analysis, or direct enzyme analysis. Duplicate samples are stored frozen at −20°C with a desiccating agent.

#### 2.3.2 Specimen preparation and LC-MS/MS analysis

The Neobase2 TM Non-derivatized MSMS kit (PerkinElmer, Wallac Oy, Turku, Finland) is used to quantify 15 amino acids, free carnitine, 35 acylcarnitines, and succinylacetone in dried blood spots according to the manufacturer’s instructions.

The vial with lyophilized isotope-labeled internal standards (IS) is recovered by adding 1.4 mL of the extraction solution that is included in the Neobase 2 kit. The Extraction Working Solution (EWS) IS prepared by diluting the recovered internal standards with the extraction solution 1:100 (v/v) and 1: 50 (v/v) for SUAC.

Dried blood samples are analyzed using Shimadzu LCMS-8050 Triple Quadrupole Mass Spectrometer (Shimadzu Corporation, Kyoto, Japan). Level I and Level II (low standard and high standard) dried blood drops are included in each assay lot of the Neobase2 TM Non-derivatized MSMS kit to monitor system accuracy and precision.

To analyze amino acids, acylcarnitines, and succinylacetone, stored DBS card samples must be brought to room temperature (+18 to +25°C) before extraction. A 3.2 mm disc (equivalent to ∼3.1 µL of whole blood) is punched out of one dried blood spot with a diameter of 3.2 mm using a Wallac DBS Puncher (PerkinElmer, Wallac Oy, Mustionkatu 6, FI-20750 Turku, Finland) into the well of the 96-well polystyrene U-bottom microplate supplied with the Neobase2 TM Non-derivatized MSMS kit. After adding 125 μL of working extraction solution to each well of the microplate, the plate is covered with an adhesive aluminum film and incubated for 30 min at room temperature on a microplate shaker with a shaking speed of 650 rpm. After incubation, 100 μL of the supernatant is transferred to a new 96-well U-bottom microplate, covered with aluminum foil to reduce evaporation and incubated for 1 h. Then, the plate is placed into the Shimadzu LCMS-8050 Triple Quadrupole Mass Spectrometer autosampler, and 5 μL of supernatant is injected into the LCMS for analysis.

Liquid chromatograph-mass spectrometer Shimadzu LCMS-8050 (Shimadzu Corporation, Japan) used for MS/MS analysis is equipped with a binary pump, an autosampler, and an electrospray ionization source (ESI). Samples are introduced into the LC-MS/MS system through an ESI source for atmospheric pressure ionization, and analysis of the samples is performed using electrospray ionization flow analysis and tandem mass spectrometry (FIA-ESI-MS/MS).

#### 2.3.3 Data acquisition and processing

A program for the LC-MS/MS solvent pump (included) is being set to deliver a mobile phase at a constant flow rate of 150 *μ*L/min, and data are collected in Positive Ion Multiple Reaction Monitoring (MRM) mode. LC-MS/MS ionization source parameters: interface voltage 4.5 kV, interface temperature 250°C, dissolution line temperature 250°C, heating block temperature 400°C, atomizing gas flow 3.0 L/min, and drying gas flow 15.0 L/min. Argon is used at a pressure of 230 kPa as a collision gas. Lab Solution software (version 5.82 SP1, Shimadzu Corporation, Kyoto, Japan) is applied for data collection. Amino acid and acylcarnitine levels will be automatically calculated according to the set internal standards using the Neonatal Solution software (version 2.20, Shimadzu Corporation, Kyoto, Japan). The total running time for each sample is 1.5 min; data is recorded within 0.9 min. The quality control samples supplied with the Neobase2 TM Non-derivatized MSMS kit are extracted and analyzed in parallel with the samples of healthy controls and patients to check the reliability of the data generated by the LC-MS/MS analysis.

#### 2.3.4 Diagnosis of targeted diseases (conditions)

The diseases to be analyzed in this study include AAD, OA, and FAOD, which are detected by measuring acylcarnitines, succinylacetone, and amino acids with MS/MS. For their diagnosis, the ratios of Phe/Tyr, C3/Met, C3/C2, C3/C0, С14:1/С2, С14:1/С16, С14:1/С12:1, С0/(С16+С18), and (C14 + C14:1 + C16:1)/C0 will be set. IEMs subject to diagnosis are displayed in Table 3. Limit values will be determined by studying levels of amino acids, acylcarnitines, and succinylacetone in DBSs of 750 healthy children in western Kazakhstan. In the case of normally distributed data, the cut-offs will be set to four standard deviations (SD) above or below the mean. If the distribution of analytes is skewed, reference intervals in the healthy control group will be determined nonparametrically and correspond to the 2.5–97.5th percentile of the experimental distribution. All cut-offs are subject to adjustment in light of further analyzes and additional clinical data.

**TABLE 3:** Primary and repeated LC-MS/MS screening and confirmatory IEM tests.

Disorder	Primary and repeated LC-MS/MS screening	Confirmatory tests
AMINO ACIDS & UREA CYCLE DISORDERS		
PKU	↑Phe, ↑Phe/Tyr ratio	↑Phe, Phe/Tyr ratio (Blood)
		↑Phenyl acetic acid (Urine)
		Genetic sequencing of the PAH gene
MSUD	↑Val, Ile, Leu	↑Val, Ile, Leu (Blood)
	↑ Leu/Ile	↑Val, Leu/Ile, Leu/Phe ratio (Blood)
	↑Leu/Phe	↑α-KG, 2OH-IVA (Urine)
	↑Xle/Ala	Genetic sequencing of the BCKDHA, BCKDHB genes
	↑Xle/Phe	
	↑Val/Phe ratio	
HCU	↑Met, Hcy	↑Met, Hcy (Blood)
	↑Met/phe ratio	
CIT I	↑Cit	↑Cit (Blood)
		↑OA (Urine)
		Genetic sequencing of the ASS1 gene
ASA	↑Argininosuccinic acid	↑Argininosuccinic acid (Blood)
		↑Argininosuccinic acid (Urine)
		Genetic sequencing of the ASL gene
OTCD	↓Cit ↑Orn	↓Cit (Blood)
		↑ Glu (Blood)
		↑Orotic acid (Urine)
		Genetic sequencing of the OTC gene
CPS	↓Cit	↑Organic acids (Urine)
		Genetic sequencing of the CPSI gene
NAGSD	↓Cit	↓Cit (Blood)
		Genetic sequencing of the NAGS gene
TYR I	↑Tyr ↑Tyr/Phe ratio	↑ Cit (Blood)
		↑ SuAc (Urine)
		↑ OA (Urine)
		Genetic sequencing of the FAH gene
TYR II	↑Tyr	Normal levels succinylacetone (Urine)
		Genetic sequencing of the TAT gene
NKHG	↑Gly	↑Gly (Blood)
		↑Gly (CSF/P)
ARG	High arginine ↑Arg/Orn	High plasma arginine and high urinary orotic acid
ORGANIC ACID DISORDERS		
SBCAD	↑С5	Genetic sequencing of the ACADSB gene
MMA	↑C3	↑C3, C3/C2 ratio (Blood)
	↑C3/C2 ratio	↑MMA, 3OH-PA, MCA, PG (Urine)
	↑C4DC, C3/C0	
PA	↑C3	↑Gly (Blood)
	↑C3/C2 ratio	↑C3, C3/C2 ratio (Blood)
	↑C3/C0 ratio	↑3OH-PA, MCA, PG (Urine)
		Genetic sequencing of the PCCA, PCCB genes
IVA	↑C5	↑C5 (Blood)
	↑C5/C2 ratio	↑IVG isovalerylglycine, ↑ 3OH-IVA (Urine)
	↑ C3/C2 ratio	Genetic sequencing of the IVD gene
IBDD	↑С4	↑С4 (Blood)
		Genetic sequencing of the ACAD8 gene
GA I	↑C5DC	↑C5-DC (Blood))
	↑C5DC/C8 ratio	↑GA, 3OH-GA (Urine)
		Genetic sequencing of the GCDH gene
3MCCD	↑C5-OH	↑C5-OH (Blood)
		↑3OH-IVA, 3MCG (Urine)
MCD, MCC	↑C3, C5-OH	↑C3, C5-OH (Blood)
		↑C3, C5-OH (Urine)
		Genetic sequencing of the HLCS gene
BTD	↑C5OH	↓Biotinidase activity (Blood) Genetic sequencing of the BTD gene
MA	↑C3DC	↑C3DC (Urine)
	↑C3DC/C4 ratio	Genetic sequencing of the MLYCD gene
MATD	↑C5:1, C5-OH	↑ C5:1, C5-OH (Blood)
		↑ TGL (tiglylglycine), 2methyl-3OH butyric (Urine)
		Genetic sequencing of the ACAT1 gene
MHBDD	↑C5	Genetic sequencing of the ACADSB gene
3HMG	↑ C5-OH	Genetic sequencing of the HMGCL gene
	↑C 5OH/C3	
	↑C6DC	
3MGA	↑C5-OH	↑C5-OH (Blood)
	↑С6DC	↑3OH-IVA, 3MGA (Urine)
		Genetic sequencing of the AUH gene
FATTY ACID OXIDATION DISORDERS		
MCAD	↑C6, C8, C10	↑n-hexanoylglycine and 3-phenylpropionylglycine (Urine) Genetic sequencing of the ACADM gene
	↑C8/C10 ratio	
	↑C10:1 ratio	
	↑ C8/C2 ratio	
VLCAD	↑C12, C14, C14:1, C16, C16:1, C18:1	Genetic sequencing of the ACADVL gene
	↑С14:1/С2 ratio	
	↑С14:1/С12:1 ratio	
	↑C14:1/C16 ratio	
SCAD	↑C3, C4, C6	↑Ethylmalonic acid (EMA) (Urine) Genetic sequencing of the ACADS gene
	↑C4	
	↑C4/C2	
LCHAD	↑C16OH	Genetic sequencing of the HADHA gene
	↑ С16OH	
	↑C18OH	
	↑C18:1OH	
MADD (GA II)	↑C4, C5, C5-OH, C5-DC, C6, C8, C14, C16, C12	↑C4, C5, C5-OH, C5-DC, C6, C8 (Blood)
		↑GA, EMA, C4–C8 AGs, isovalerylglycine (Urine)
		Genetic sequencing of ETFA, ETFB, ETFDH genes
CUD	↓C0	↓C0 (Blood)
	↓long-chain acylcarnitines	↓C0 (Urine)
		Genetic sequencing of the SLC22A5 gene
CPT I	↑C0, ↑C0/C16 + C18 ratio	↑C0 (Blood))
	↓C16, C18, С18:1, С18:2	↑C0/C16 + C18 (Blood)
		Genetic sequencing of CPT1A, CPT1B, CPT1C genes
CPT II	↓C0	Genetic sequencing of the CPT2 gene
	↑C14, C16, C16:1, C18, C18:1, С18:2	
CACT	↓C0	Genetic sequencing of the SLC25A20 gene
	↑C16, C18, C18:1, С18:2	
DE RED	↑С10:2	Genetic sequencing of the DECR1 gene
MCAT	↑C6-OH	
	↑C8	
	↑С10:1ОН	

#### 2.3.5 Confirmatory tests

Patients will be immediately referred for confirmatory tests if the results exceed the thresholds. A reanalysis of the same sample will be performed if the results fall outside the threshold. Patients will be referred for confirmatory tests if the second test also falls outside the threshold.

Confirmatory tests include:

Clinical data; analysis of therapeutic effects; reanalysis LC-MS/MS; urine analysis for organic acids by gas chromatography-mass spectrometry (GC-MS); amino acid analysis; routine blood test; blood biochemistry; blood gas analysis; tests for glucose and ammonia in the blood; tests for homocysteine, lactate and pyruvate in the blood; tests for acetone in the urine; biotin, biotin enzyme profile; measurement of enzyme activity; tests for gene mutations; DNA analysis; magnetic resonance imaging of the brain and electroencephalography.

As known, gas chromatography-mass spectrometry is one of the best methods for diagnosing congenital acidemia. As organic acids are practically not reabsorbed in the renal tubules, their concentration in urine is higher than in the blood, and they are easier to determine in urine. Patients with a positive dry blood spot test result using TMS with or without clinical manifestations of IEM will have urine organic acids determined by gas chromatography-mass spectrometry.

To determine organic acids in urine using GC-MS, collecting 10–15 mL of morning urine in a clean, dry container without preservatives and transporting it in a thermos with food ice is necessary. The urine can be frozen and stored at a temperature of −20 C0 until analysis (Gallagher et al., 2018).

Before collecting urine, appropriate instructions regarding fluid intake, consumption of certain foods, avoidance of certain medications, etc., should be followed.

Several protocols have been developed for analyzing organic acids in urine samples in recent years. Typically, each of them includes five steps:

1. Oximation by exposure to hydroxylamine. This step, although optional, is required to stabilize 2-keto acids, thus allowing a better recovery.
2. Organic acids separation by liquid–liquid extraction.
3. Trimethylsilyl (TMS) derivatization of the dried samples, obtained by adding BSTFA (N,Obis (trimethylsilyl)trifluoroacetamide): It is performed to improve the thermal stability and volatility of the organic acids.
4. Injection into the GC/MS (Gas Chromatograph/Mass Spectrometer) apparatus. Scanning the resulting ions based on different mass/charge values allows a fragmentation profile (mass spectrum) to be constructed.
5. Identification of mass spectra. The analysis is carried out on a tandem quadripole time-of-flight gas chromatograph—mass spectrometer “Agilent 7500 Q-TOF” to determine the concentration of the corresponding organic acid in urine according to the obtained mass spectrogram. The concentration of a specific organic acid in the urine is calculated as mmol/mol creatinine (in terms of normal creatinine) (Villani et al., 2017).

Identifying the affected individual has important implications for other family members, who may also require TMS dry blood spot testing, urine organic acid testing, and additional genetic testing.

Genetic counseling:

Aminoacidopathies, organic aciduria, and disorders of fatty acid metabolism are primarily inherited in an autosomal recessive manner. The parents are obligate heterozygotes; accordingly, each has one copy of the mutation that causes this disease. Heterozygous carriers have no symptoms of the disease. At conception, each sibling of the proband has a 25% risk of being affected, and in 50% of cases, being an asymptomatic carrier, a 25% chance that the child will not be sick and will not be a carrier. Unaffected siblings of affected individuals have a 2/3 chance of being heterozygotes.

DNA diagnostics. Next-Generation Sequencing:

Dried blood spots or whole blood can be used for whole exome sequencing. When using whole blood: collect 2–5 mL of the patient’s blood into tubes with EDTA preservative (purple cap); carefully invert the tubes several times to mix with the preservative, but do not shake to avoid hemolysis. Transport in a thermos with food ice can be frozen and stored at a temperature of - 80 C0. Whole blood must be delivered no later than 7 days after collection.

To conduct whole-exome sequencing of human DNA using NGS technology, sequencing platforms, a sequencer, an automatic station for DNA extraction, a fluorimeter, a centrifuge, a laminar flow cabinet, a flow-through bactericidal air recirculation, a fridge, nucleic acid quantitation instrument; nucleic acid quality analyzer; thermal cycler; vortex spot; ultrasonicator; standard laboratory supplies (pipettes, 96-well plates, centrifuge tubes) are needed.

This procedure is carried out by the institution’s approved standard operating procedures and the NGS protocol guidelines of the technology used and includes three stages:

Stage I: Preparation of the library. Preparing the library consists of the following steps: DNA sample extraction, PCR amplification of DNA, and library cleaning.
Stage II: Sequencing: reading the obtained information from the biochip and transferring the data to the software. Studies use the NovaSeq™ 6,000 Sequencing System (Illumina, Inc.).
Stage III: Bioinformation analysis: a bioinformatic analysis of the obtained data and laboratory interpretation of the results are being carried out (Fernandez-Marmiesse et al., 2018; Marshall et al., 2020; Austin-Tse et al., 2022).

Table 3 presents the criteria for primary and repeated LC-MS/MS screening for IEM and confirmatory tests.

The tests will be conducted in the INVITRO laboratory (Kazakhstan, Almaty) by ICH E-6 Guidelines for Good Clinical Practice. The INVITRO laboratory is accredited to determine the profiles of acylcarnitines, succinylacetone, and amino acids by LS-MS/MS analysis by ISO 15189. From October 2022 to August 2024, the frequency of 37 AAD, OA, and FAOD will be assessed using LC-MS/MS technology in a group of children at high risk. A summary report on screening high-risk children for IEM in western Kazakhstan by LC-MS/MS will be presented.

## 3 Presenting the anticipated results

### 3.1 Study outcomes

The definition applied in the present study is as follows: Inborn errors of metabolism are inherited disorders caused by mutations in genes coding for proteins that function in metabolism. In particular: Amino Acids and Urea Cycle Disorders, Organic Acid Disorders, and Fatty Acid Oxidation Disorders.

Accordingly, we apply the following definitions of measured outcomes.

• Primary outcomes:

1. Normal and abnormal values of amino acids, acylcarnitines, and succinylacetone in dry blood spots of newborns and children of different age groups measured in µmol/L through liquid chromatography-tandem mass spectrometry (LC-MS/MS).

Metabolites to be measured:

Amino acids: Alanine (Ala), Arginine (Arg), Citrulline (Cit), Glutamine (Gln), Glutamic acid (Glu), Glycine (Gly), Leucine (Leu), Isoleucine (Ile), Hydroxyproline (Pro-OH), Methionine (Met), Ornithine (Orn), Phenylalanine (Phe), Proline (Pro), Tyrosine (Tyr), Valine (Val). Acylcarnitines: Free carnitine (C0), Acetylcarnitine (C2), Propionylcarnitine (C3), Malonylcarnitine+3-Hydroxybutyrylcarnitine (C3DC/C4OH), Butyrylcarnitine (C4), 2H9-C5-Methylmalonylcarnitine+3-Hydroxyisovalerylcarnitine (C4DC/C5OH), Isovalerylcarnitine (C5), Tiglylcarnitine (C5:1), Glutarylcarnitine (C5DC), Hexanoylcarnitine (C6), Octanoylcarnitine (C8), Octenoylcarnitine (C8:1), Decanoylcarnitine (C10), Decenoylcarnitine (C10:1), Decadienoylcarnitine (C10:2), Dodecanoylcarnitine (C12), Hydroxydodecenoylcarnitine (C12:1), Myristoylcarnitine (C14), Tetradecenoylcarnitine (C14:1), Tetradecadienyl-carnitine (C14:2), Hydroxytetradecanoylcarnitine (C14OH), Palmitoylcarnitine (C16), Hexadecenoylcarnitine (C16:1), 2H3-C16-3-Hydroxy-Hexadecanoylcarnitine (C16OH), 2H3-C16-3-Hydroxypalmitoleylcarnitine (C16:1OH), 2H3-Stearoylcarnitin (C18), 2H3-C18-Octadecenoylcarnitine (C18:1), 2H3-C18-Linoleylcarnitine (C18:2), 2H3-C18-3-Hydroxystearoylcarnitine (C18OH), 2H3-C18-3-Hydroxyoleoylcarnitine (C18:1OH), 2H3-C18-3-Hydroxylinoleoylcarnitine (C18:2OH). Succinylacetone (SUAC) (13C5-MPP IS).

2. The number and proportion of children with detected abnormal metabolite values and checked by retesting and/or confirmatory tests if the results exceeded the thresholds.

• Surrogate endpoints:

1. Abnormal values of organic acids in urine measured through gas chromatography-mass spectrometry (GC-MS) applied for confirmatory tests.
2. Abnormal values of any other tests, such as tests for glucose and ammonia in the blood; tests for homocysteine, lactate, and pyruvate in the blood; tests for acetone in the urine; biotin, biotin enzyme profile; measurement of enzyme activities; tests for gene mutations; DNA analysis; magnetic resonance imaging of the brain and electroencephalography.
3. The number, combination, and severity of clinical symptoms in high-risk children confirmed by the pediatricians’ examination.

### 3.2 Statistical analysis

Shapiro-Wilk and Kolmogorov-Smirnov tests will be used to check the normality of the distribution. As a rule, the distribution of free carnitine and acylcarnitines concentrations in whole blood differs from normal (Manta-Vogli et al., 2020). If the data sets show a normal distribution, M (mean) and standard deviation (error of the mean) will be used for descriptive statistics of the samples. Parametric tests will be used to test for differences between AA and AC concentrations depending on various factors (gender, place of residence) (t-test, analysis of variance (ANOVA) with or without Tukey’s test). If the data sets do not show a normal distribution, Me (median) and quartiles (interquartile range IQR) will be used for the descriptive statistics of the samples. Non-parametric tests (Mann-Whitney U-test, Kruskal-Wallis H-test without or with subsequent *post hoc* comparisons using U-test will be applied to test the differences mentioned above. The Pearson χ^2^ criterion will be applied to identify intergroup differences for categorical variables. If the distribution of analytes is skewed, reference intervals in the healthy control group will be determined nonparametrically and correspond to the 0.5–99.5th percentile of the experimental distribution. Correlations between body weight, and concentration of each analyte will be performed using corresponding tests.

Indices of sensitivity (Sn), specificity (Sp), positive predictive values (PPVs), and negative predictive values (PVN) will be calculated for each diagnostic test under consideration. PPVs will be calculated by dividing the total number of true positive cases by the total number of true positive and false positive cases. The detection rate will be calculated by dividing the number of cases diagnosed by the number of screened children with suspected metabolic disorders. Multiple linear regression coefficients will be calculated to assess the relationship of each analyte with the factors of gender, weight, and place of residence. Due to the number of variables subjected to analysis, and the anticipated presence of multiple outcomes, the included tests may not be sufficient. So, the cluster analysis and, possibly, the decision-tree method will be applied. Decision trees break down complex data into more manageable parts, beneficial in prediction analysis, data classification, and regression. A dimensionality reduction method, principal component analysis (PCA), will also be applied. Two-sided levels <0.05 are assumed to be statistically significant. Statistical analysis will be carried out using the statistical packages IBM SPSS v. 23.0 (IBM, Armonk, NY, United States), Statistica (StatSoft, Inc., Tulsa, OK, United States, v. 10), and R 3.3.2 (R Foundation for Statistical Computing, Vienna, Austria.)

#### 3.2.1 Descriptive statistics and group comparison

Descriptive statistics and reference intervals for whole blood concentrations of 15 AAs, 35 ACs, and SuAc in healthy children divided into subgroups according to gender will be presented in tables. For each analyte in the healthy control group, the upper cut-off will be set above the 97.5^th^ percentile, while the lower cut-off will be set below the 2.5th percentile. Differences in the distribution of amino acids, acylcarnitines, and succinylacetone between groups, as determined using the Mann-Whitney and Kruskal-Wallis tests, will be noted in the table if found.

Cut-off values for analytes and their ratios for selective screening of AAD, OA, and FAOD presented in Table 3 will be pointed out. The results of determining the analytes in DBS in children of the healthy control group will be compared with those of previously published studies in other populations. According to the inclusion criteria, healthy newborns in the reference group must be full-term (born at 37–42 weeks). However, it should be taken into account that some of the newborns at risk for IEM are born prematurely. In this regard, it is also necessary to determine reference intervals for the concentrations of 15 AAs, 35 ACs and SuAc in dried blood spots of premature infants who do not have clinical symptoms of IEM.

Comparison of mean concentrations (Me, range) of analytes between groups of high-risk children and healthy controls will be presented.

#### 3.2.2 Statistical analysis by gender

The statistical analysis by gender (Mann-Whitney U-test) will be presented in groups of high-risk and healthy children. Comparison results by analytes taking into account gender, and body weight in the selective screening group and the group of healthy children will be presented in tables. In the presence of significant differences, in the form of box plots, where rectangles correspond to the 25th and 75th percentiles, the central and horizontal line is the median for each analyte. For all analytes, body weight scatterplots will be presented with a mean (M), median (Me), percentiles, and regression lines. Each analyte has its scale, measured in µmol/L.

### 3.3 Presenting detected cases

The results of LC-MS/MS in affected individuals will be presented as metabolic profiles. The metabolic profile of each affected individual will be placed next to the metabolic profile of a healthy individual from the control group, corresponding to gender, age, and place of residence. IEMs detected, their frequency, primary clinical manifestations, and main diagnostic markers in high-risk children confirmed by LC-MS/MS will be described in the tables. The average age of patients at the time of diagnosis will also be recorded. Table 4 displays the most common clinical manifestations in patients diagnosed with IEM. This list can be expanded by the results obtained in the study.

**TABLE 4:** Common features encountered in children with different IEM (N = ***).

Variables	Number of patients (percent)
Developmental delay	
Neurological abnormalities	
Disturbed consciousness level	
Vomiting/dehydration	
Hyperammonemia	
Metabolic acidosis	
MRI brain abnormalities	
Infections	
Hypoglycemia	
Mental retardation	
Organomegaly	
Micro/macrocephaly	
Ophthalmic abnormalities	
Cardiomyopathy	
Seizures	
Tachypnea	
Thrombocytopenia	
Abnormal smell	
Hair growth disorders	
Osteoarticular anomalies	
Abnormal muscle tone	
Mutism	
Dysmorphia	

The baseline and repeated LC-MS/MS analysis results and biochemical confirmation in children diagnosed with IEM, according to the diagnosed disease, will also be presented. Demographic and laboratory data of confirmed cases detected by LC-MS/MS among 1,500 Kazakhstani children with suspected IEM will be presented in Table 5. Also, the table will include data on the reference values of the considered metabolites obtained in a sample of healthy controls.

**TABLE 5:** Cases detected by LC-MS/MS among 1,500 children suspected IEM.

Metabolic disorder	Metabolite detected by LC- MS/MS (Abnormal parameter)	Positive cases, n (%)	Concentration mean (range) (μmol/L)	Cut-off limit (μmol/L)	References range (μmol/L)	Province (oblast), positive cases (n)
						
						

IEM, identified by selective screening with calculated frequencies for each disease, will be described separately for diseases belonging to the AAD, OA, and FAOD groups.

### 3.4 Presenting IEM frequencies in the child population

The frequency of inborn errors of metabolism detected using tandem mass spectrometry, depending on the geographical location and type of IEM in western Kazakhstan during the period from 2023 to 2024, can be represented graphically in the form of accumulation diagrams demonstrating the contribution of AAD, OA, and FAOD to the total incidence of IEM. Also, these data can be presented in the form of a table. The data will include the total number of IEM cases in the high-risk group of children and the absolute number and frequency of cases for each identified disease. Detection rates for selective screening and incidence will be calculated according to the number of children born in western Kazakhstan during the study period, in 2023–2024, for each specific disease. These data will allow for estimating the number of affected male and female children and determining which IEMs were the most common in the studied population. Given that selective screening of the child population in western Kazakhstan will be carried out for about 2 years (2023–2024, recruitment of high-risk children), the incidence rate for each class of disorders (AAD, OA, FAOD) will be established, taking into account the total number of births each year.

### 3.5 Presenting positive and false positive cases

Even though the number of false positive results during LC-MS/MS is much lower than when using standard biochemical tests and, according to different researchers, ranges from 1.9% to 2.3% (Selim et al., 2014; Hassan et al., 2016), we assume that a certain number of samples will turn out to be a false positive. The number of true and false positives for each disease according to each disorder after the primary (baseline) LC-MS/MS will be established. Positive predictive values will be calculated by the total number of true positive cases divided by the total number of true positive and false positive cases.

## 4 Discussion

The present study assumes only selective screening for IEM. Implementing ENBS, which allows for thoroughly assessing the prevalence of IEM in the child population of western Kazakhstan, is currently unavailable due to a lack of proper financing. In addition, given that the overall prevalence of IEM in populations is relatively low, less than 1:100,000 for most of them, coverage for expanded newborn screening should be total. Besides, it is known that not all IEMs are diagnosed during ENBS, as some disorders debut at a later age (Hassan et al., 2016). Nonetheless, the data of selective screening conducted among children at high risk of IEM, given the inclusion in the study of the largest pediatric hospitals in western Kazakhstan, can serve as a basis for calculating the relative frequencies of various IEMs in the child population of the region.

Detection rates for selective screening and incidence will be calculated according to the number of children born alive in the region during the study period, in 2023–2024, for each specific disease. These data will allow for estimating the number of affected male and female children and determining which IEMs are the most common. The frequencies of each IEM revealed across the child population of western Kazakhstan will be matched with incidence data in other regions of the world.

Conducting ENBS and selective screening for IEM in Kazakhstan is of particular interest due to the entire absence of similar studies in the region of Central Asia, despite a significant number of studies conducted in neighboring areas (Russia, China, India, the Middle East, countries of East and Southeast Asia) (Guthrie and Susi, 1963; Nagaraja et al., 2010; Shibata et al., 2018; Therrell and Padilla, 2018; Yang et al., 2020; Deng et al., 2021; Loeber et al., 2021). A study by Shibata et al. revealed different rates of IEM incidence and spectrum of diseases in Asian countries. In addition, it was found that the ranges of IEM diseases determined using selective screening differed from those detected using ENBS (Shibata et al., 2018). For instance, the incidence of phenylketonuria in Asians (∼1:50,000 in Korea; ∼1:110,000 in Japan) is much lower than in Caucasians (∼1:10,000). The medium-chain acyl-CoA dehydrogenase (MCAD) deficiency, a common genetic metabolic disorder in Caucasian children with the A985G mutation of frequent occurrence, can serve as another example. Unlike Caucasians, MCAD is extremely rare in Korea and Japan (Vieira Neto et al., 2012). The frequencies (specific contribution to the IEM spectrum) of FAOD in the United States newborn population are, according to various studies, 45.2% in North Carolina (Frazier et al., 2006) and 38% in California (Feuchtbaum et al., 2012), which is slightly higher than in some European countries (Lindner et al., 2011; Shibata et al., 2018) and much exceeds the frequency of FAOD in Asian countries (Guthrie and Susi, 1963; Golbahar et al., 2013; Selim et al., 2014; Hassan et al., 2016; Shibata et al., 2018). FAOD detection rates for selective screening vary across countries, ranging from 0.29% in a South Korean study (Yoon et al., 2005) to 10.8% in a study in Oman (Al Riyami et al., 2012).

Thus, the number of IEMs to be included in the protocol of the future ENBS in Kazakhstan through LC-MS/MS will depend, in addition to economic factors and laboratory equipment, on the frequency of diseases established during selective screening. In describing established cases of IEM, in addition to presenting the data specified in the Results section, the present study implies reflecting the family history of IEM, including the number of patients and families with IEM, gender, positive family history, consanguineous marriages, and age of the symptoms onset. Although, due to the historically and socially established forbiddance for consanguineous marriages in Kazakhstan, we assume their rare prevalence, the data obtained would be of interest for comparison with populations for which similar results are already available (Guthrie and Susi, 1963; Al Riyami et al., 2012; Golbahar et al., 2013; Hassan et al., 2016; Gayduk et al., 2022).

In the present study, the distribution of IEM primary clinical symptoms and their severity will be analyzed by age. Delayed diagnosis at an older age is commonly associated with many severe and irreversible clinical complications in patients (Champion, 2010; Selim et al., 2014; Han et al., 2015; Lampret et al., 2015; Dietzen et al., 2016). This circumstance calls for the urgent need for early detection and intervention to achieve favorable outcomes in IEM.

• Study limitations.

1. The number of children with clinical manifestations to be examined in the ongoing screening for IEM by LC-MS/MS is limited.
2. Other parts of Kazakhstan are not involved in the study. As such, assessing the differences between the levels of AAs, ACs, and SuAc in DBS of the child population and the differences in the IEM incidence appear impossible.
3. The need for multiple and expensive tests to rule out rare and ultra-rare IEM.
4. Metabolites for diagnosing other hereditary metabolic diseases, such as disorders of carbohydrate metabolism, disorders of porphyrin metabolism, disorders of purine and pyrimidine metabolism, disorders of steroid metabolism, disorders of mitochondrial function, disorders of peroxisomal function, and lysosomal storage disorders, are not included in this study.

As such, this study is the first in Kazakhstan and Central Asia to assess the prevalence of IEM among children at risk. Also, reference values of metabolites for three different age groups of the child population in western Kazakhstan will be established for the first time. Our research can be considered a pilot and expected to give an impulse to launch a nationwide ENBS program using LC-MS/MS. Overall, the study will further develop the national as selective as expanded newborn screening programs.

## 5 Ethics and dissemination

This research poses no risk to participating individuals. Study participation does not imply restrictions in any clinical care determined by pediatricians. All study procedures are conducted according to the principles of the Declaration of Helsinki (2013), and patient rights are observed. Participation in the study is voluntary. Informed consent is obtained from all parents and/or legal guardians of children involved in the study. Written informed consent is obtained from the patients’parents and/or legal guardians to publish all subsequent papers under the following conditions: their data must be placed anonymously in the research database without mentioning personal details, i.e., 600 under coded numbers. The study was approved by the Bioethics Committee of the West Kazakhstan Marat Ospanov Medical University (Ref.: No. 7.04, 29/09/2022.)

The dissemination plan includes publishing two articles, not accounting for the Study Protocol. The first paper will report scientific results on set reference values in the group of healthy controls, and the subsequent publication present the research’s final results (in peer-reviewed journals). The dissemination plan does not imply but makes it possible to develop national-scale clinical recommendations to manage IEM in the country.

## Figures and Tables

**Table:** 

**3HMG**	3 hydroxy methylglutaryl-CoA lyase deficiency, 3-hydroxy 3-methyl glutaric aciduria
**3MCCD**	3-methylcrotonyl-CoA carboxylase deficiency
**3MGA**	3-methylglutaconyl-CoA hydratase deficiency, 3-methylglutaconic acid/aciduria
**AAD**	amino acid disorders
**AAs**	amino acids
**ACs**	acylcarnitines
**Ala**	alanine
**Arg**	arginine
**ARG**	argininemia, arginase deficiency
**ASA**	Argininosuccinic aciduria
**BTD**	biotinidase deficiency
**C0**	free carnitine
**C2**	acetylcarnitine
**C3**	propionylcarnitine
**C3DC**	malonylcarnitine
**C4**	iso-/butyrylcarnitine
**C4DC**	methylmalonylcarnitine
**C4OH**	3-hydroxyiso-/butyrylcarnitine
**C5 OH**	hydroxyisovalerylcarnitine
**C5**	isovaleryl-/2-methylbutyrylcarnitine
**C5:1**	tiglylcarnitine
**C5DC**	glutarylcarnitine
**C6**	hexanoylcarnitine
**C6DC**	3-methylglutarylcarnitine
**C6OH**	3-hydroxyhexanoylcarnitine
**C8**	octanoylcarnitine
**C8:1**	octenoylcarnitine
**C8DC**	octanedioylcarnitine
**C10**	decanoylcarnitine
**C10:1**	decenoylcarnitine
**C 10:2**	decadienoylcarnitine
**C10DC**	sebacylсarnitine
**C12**	dodecanoylcarnitine
**C12:1**	dodecenoylcarnitine
**C12DC**	dodecanedioylсarnitine
**C14**	tetradecanoylcarnitine
**C14OH**	3-hydroxytetradecanoylcarnitine
**C14:1**	tetradecenoylcarnitine
**C14:2**	tetradecadienoylcarnitine
**C16**	hexadecanoylcarnitine
**C16OH**	3-hydroxyhexadecenoylcarnitine
**C16:1**	hexadecenoylcarnitine
**C16:1OH**	3-hydroxyhexadecenoylcarnitine
**C18**	octadecanoylcarnitine
**C18OH**	3-hydroxyoctadecanoylcarnitine
**C18:1**	octadecenoylcarnitine
**C18:1OH**	hydroxyoleoylcarnitine
**C18:2**	linoleylcarnitine
**C18:2OH**	3-hydroxyoctadecadienoylcarnitine
**CACT**	carnitine/acylcarnitine translocase deficiency
**CIT I**	citrullinemia I
**Cit**	citrulline
**CPS**	carbamoyl phosphate synthetase deficiency
**CPT I**	carnitine palmitoyl transferase deficiency type I
**CPT II**	carnitine palmitoyl transferase deficiency type II
**CUD**	carnitine uptake defect
**DBS**	dry blood spot
**DE RED**	2,4 dienoyl-CoA reductase deficiency
**ENBS**	expanded newborn screening
**FAOD**	fatty acid oxidation defects
**GA I**	glutaric aciduria type I, glutaryl-CoA dehydrogenase deficiency
**GC-MS**	gas chromatography-mass spectrometry
**Gln**	glutamine
**Glu**	glutamic acid
**Gly**	glycine
**HCU**	homocystinuria
**IBDD**	isobutyryl-CoA dehydrogenase deficiency
**IEM**	inborn errors of metabolism
**Ile**	isoleucine
**IMD**	inherited metabolic diseases
**IVA**	isovaleric academia, isovaleryl-CoA dehydrogenase deficiency
**LCHAD**	long-chain 3-hydroxyacyl-CoA dehydrogenase deficiency, trifunctional protein deficiency
**LC-MS/MS**	liquid chromatography-tandem mass spectrometry
**Leu**	leucine
**MA**	malonic acidemia, malonyl-CoA decarboxylase deficiency
**MADD (GA II)**	multiple-CoA dehydrogenase deficiency, glutaric acidemia type II, glutaryl-CoA dehydrogenase type II deficiency
**MATD**	mitochondrial acetoacetyl-CoA thiolase (T2) deficiency/“beta-ketothiolase”
**MCAD**	medium-chain acyl-coenzyme A dehydrogenase deficiency
**MCAT**	medium-chain ketoacyl-CoA thiolase deficiency
**MCD**	multiple carboxylase deficiency
**Met**	methionine
**MHBDD**	2-methyl-3-hydroxybutyryl-CoA dehydrogenase deficiency
**MMA**	methylmalonic academia, methylmalonyl-CoA mutase deficiency
**MSUD**	maple syrup urine disease
**NAGSD**	N-acetylglutamate synthase deficiency
**NBS**	newborn screening
**NKHG**	nonketotic hyperglycinemia
**OA**	organic acidemias
**Orn**	ornithine
**OTCD**	ornithine transcarbamylase deficiency
**PA**	propionic acidemia, propionyl-CoA carboxylase deficiency
**Phe**	phenylalanine
**PKU**	phenylketonuria
**Pro**	proline
**Pro-OH**	hydroxyproline
**SBCAD**	short/branched chain acyl-CoA dehydrogenase deficiency, **2-**methylbutyryl-CoA dehydrogenase deficiency
**SCAD**	short-chain hydroxyacyl-CoA dehydrogenase deficiency
**SuAc (SUAC**	succinylacetone
**TYR I**	tyrosinemia type I
**TYR II**	tyrosinemia type II
**Tyr**	Tyrosine
**UCD**	urea cycle disorders
**Val**	valine
**VLCAD**	very long chain acyl-CoA dehydrogenase deficiency
